# Implementation of a Universal Framework Using Design Patterns for Application Development on Microcontrollers

**DOI:** 10.3390/s24103116

**Published:** 2024-05-14

**Authors:** Marek Babiuch, Petr Foltynek

**Affiliations:** Department of Control Systems and Instrumentation, VSB—Technical University of Ostrava, 70800 Ostrava, Czech Republic; petr.foltynek.st@vsb.cz

**Keywords:** design pattern, framework, IoT, microcontroller, programming, SOLID

## Abstract

This article focuses on the area of software development for microcontrollers and details the implementation of modern programming practices and principles in embedded systems and IoT applications. This article explains how we implemented previously unimplemented principles and applied design patterns for quality software design on microcontrollers, which are currently only used for developing applications on the higher layers of the IoT reference model. A custom modular framework for microcontrollers is presented, based on applying SOLID principles and adapting design patterns specific to the microcontrollers’ application development needs. The implemented framework enables independent communication between modules and flexible integration of hardware components. It is designed with platform independence in mind, contributing to its wide adaptability and ease of use in diverse development environments. By applying these technological approaches, we can create applications that are not only testable and extensible in terms of application logic but also allow for easy adaptation to changes in these hardware resources. Utilizing these capabilities represents an innovative approach to development for microcontrollers that fundamentally improves the long-term sustainability and scalability of applications.

## 1. Introduction

Our goal was to create an innovative development approach for microcontrollers that fundamentally increases applications’ long-term sustainability and extensibility. This approach extends the life of the developed systems and significantly reduces the costs of their maintenance and further development, bringing about revolutionary changes in the way IoT systems and applications are designed and managed. From our detailed survey, no other work has dealt with a similar specific application of SOLID principles, design patterns, and modular framework design for microcontroller applications, highlighting this innovation’s unique nature and potential impact on the embedded-system application development field. This article details the steps in developing a modular framework for microcontrollers that enables developers to create flexible and sustainable applications. This section presents the current state of technology and trends in microcontroller software development, providing readers with a context for understanding the motivation and need for the new approach. The following provides a detailed analysis of the implementation of SOLID principles in the environment of microcontrollers, emphasizing the adaptation of these principles concerning the limitations of given microcontrollers, and including the specifics of the programming languages used. Special attention is paid to implementing the principle of dependency injection without using RTTI, which is significant in limiting computing resources and ensuring compatibility with different types of microcontrollers. The following section describes the architectural design of the modular framework, which is designed to be independent of HW resources and to allow efficient communication between different modules. This design ensures the framework can adapt to various hardware changes without significant code modifications, significantly increasing its maintainability and extensibility. In the final sections, there is a critical discussion of the implemented steps and an assessment of their impact on the practical use of the framework. Possibilities and challenges for further development are discussed, emphasizing potential innovations and improvements.

Various frameworks and tools are used to develop applications for microcontrollers, with the most popular platforms representing the most accessible approach to programming different types of microcontrollers due to their open-source nature and support for many microcontrollers. These IDEs use a C-based programming language, allowing easy programming of microcontrollers without significant hardware knowledge. An alternative to these environments is MicroPython [1,2], which is optimized to run on microcontrollers and is already used as firmware on some devices. However, the most common development languages are based on C with a procedural style of writing code. It is also possible to develop applications using C++, which implements object-oriented principles, but we must be aware that we may run into the performance limits of the microcontroller. C/C++ languages are very efficient tools for developing microcontroller applications. They can be used to manage memory management very well, but we have to remember that the programmer takes care of it, which creates a significant source of errors.

In today’s practice, procedure-oriented programming is most commonly used in developing applications for microcontrollers [3]. We program using loops, branching, and decision conditions. The program can be decomposed into individual functions. The principle of procedural programming is the functional decomposition of a given problem. The problem is divided into several sub-problems, and a specific function with parameters implements each sub-problem. The disadvantage of this approach is that a given function is designed for only one activity; when we want to perform something else, we have to write a new function. A program designed in this way is difficult to maintain. Each modification of the program increases its complexity. The program gradually reaches a state where the cost of adding new functions increases so much that it is no longer worth extending. The C language or Python represents this approach.

The primary motivating factor for the emergence of the object-oriented approach is the elimination of some of the shortcomings of procedural programming. The object-oriented approach considers data as a crucial element in program development. It then binds the data to the functions that work with the data and protects them from accidental modification from an external function. The object-oriented approach allows the decomposition of the problem. The decomposition is performed in a bottom-up approach, where the basic building blocks are defined, and the whole program is only built from them. The basic building blocks are referred to as objects. The data are then bound to the objects, and the behavior of the objects is implemented by functions (also called methods). Only functions associated with an object can access the data of that object. A function of one object can access functions of other objects. This makes it possible to reuse source code. Developing a program using the object-oriented approach essentially models reality, where objects are images of real-world objects. The programs implemented using object-oriented principles provide the following benefits:

- With inheritance, we can remove redundant code and extend the use of existing code.
- Functionality and responsibilities are safely encapsulated in separate classes.
- We can create programs from standard pre-built modules that communicate with each other instead of having to start writing code from scratch. This leads to development time savings and increased productivity.
- The principle of data hiding helps the programmer to create a safe program.
- It is possible for multiple instances of the same object to exist side by side without affecting each other.
- It is possible to map objects in the problem domain to objects in the program.
- Divining the work into an object-based project among multiple programmers is easy.
- The data-centric design approach allows us to capture more system details.
- An object-oriented system can be easily upgraded from a small system to a large system.
- Message passing techniques for communication between objects allow for a more straightforward description of interfaces with external systems.
- Software complexity can be easily managed.

While all of these features can be incorporated into an object-oriented system, their importance depends on the type of project and the programmer’s preferences. The above advantages solve many existing problems that arise during code development. To avoid problems in application development, it is advisable to take full advantage of the power of the object-oriented approach, and it is also desirable to design the application using SOLID principles.

## 2. SOLID Principles and Design Patterns

Our goal is to create an implementation of the framework on microcontrollers that uses modern programming principles and design patterns described in this article. These principles and design patterns are not yet implemented on microcontrollers, even though they are commonly used in software applications at the higher layers of the IoT reference model. By implementing this framework, we will achieve cheaper application development of microcontrollers, increase the sustainability and extensibility of a given application over time, and streamline the application development cycle by reducing physical testing on a given hardware.

### 2.1. SOLID Principles

SOLID principles are a set of guidelines and practices that help us write maintainable and less error-prone applications. SOLID [4,5] is an acronym for five design principles for implementing an object-oriented approach:

- Single Responsibility Principle;
- Open–Closed Principle;
- Liskov Substitution Principle;
- Interface Segregation Principle;
- Dependency Inversion Principle.

The primary purpose of these principles is to limit the dependencies between different parts of the application and to limit the need for subsequent modifications after each change in the application. Dependencies between objects, especially hidden ones, are the main reason an application becomes unmaintainable after a certain period of time and requires significant design changes or even the creation of a new version of the application from scratch.

### 2.2. Design Patterns

The issue of design patterns is a vast science dealing with software design [6,7]. Design patterns formalize general solutions to common programming problems that often arise in software development and provide a common language for discussing and solving these problems. Applying design patterns helps developers achieve better code quality, more manageable code maintenance, and increased code reusability. Design patterns [8] can be divided into three categories depending on their purpose and function:

- Behavioral patterns focus on addressing issues related to interactions between objects and classes. These patterns include Chain of Responsibility, Command, Interpreter, Integrator, Mediator, Memento, Observer, State, Strategy, Template Method, and Visitor.
- Structural patterns focus on solving questions about organizing objects and classes into larger units. These patterns include Adapter, Bridge, Composite, Façade, Decorator, Flyweight, and Proxy.
- Creational patterns are patterns that focus on addressing issues related to object creation and class instantiation. These patterns include the abstract Factory, Builder, Factory method, Prototype, and Singleton.

### 2.3. Model-View-Controller Design Pattern

Model-View-Controller (MVC) is a fundamental design pattern in software engineering that provides a robust structure for organizing code, increasing the maintainability and extensibility of applications by separating application logic from the user interface and data representation. The MVC [9,10] structure is divided into three main components that work together to provide a coherent and efficient way of processing and presenting data. The responsibilities of each component in the MVC are as follows:

- Model: This represents the representation and management of application data, including interaction with database systems and other data sources. It includes the rules and operations that define how data can be created, retrieved, updated, and deleted.
- View: This is responsible for presenting data to the user. This may be through a graphical user interface, a text interface, or some other form of output.
- Controller: This serves as a communication intermediary between the Model and the View. It processes user input that updates the Model and displays the results to the user through the View.

The Model-View-Controller (MVC) design pattern ranks among the leading paradigms in software engineering. It explicitly segregates responsibilities between application components [11]. Its application across different technologies has led to the creation of many derivatives adapted to the specific needs of development environments. Variants in the MVC pattern include Model-View-Presenter (MVP) [12], Model-View-ViewModel (MVVM) [13,14], and Model-View-Adapter (MVA) [15], among others.

### 2.4. Dry Principle (Don’t Repeat Yourself)

The DRY principle is another principle that is used in application development. As the name suggests, it aims to minimize code duplication by encouraging the reuse of software parts through abstraction, modularization, and automation of code generation. Applying this principle in microcontroller application development can lead to more efficient memory usage and more manageable code maintenance; this is crucial in the constrained environment of microcontrollers. By introducing the DRY principle, developers can reduce redundancy, making it easier to identify and fix bugs, improve code readability, and enable the faster implementation of new features. It can also improve application performance by reducing the required instructions and freeing resources for other critical operations. The above design patterns and principles are commonly used when developing applications in higher-level programming languages such as C#, Java, Dart, or Kotlin. These languages are very suitable for creating modern frameworks. For example, Microsoft’s .NET Framework is implemented using C#, and Google’s Flutter framework is implemented using Dart; however, development for microcontrollers still heavily relies on a procedural style of programming that is difficult to test and makes the application unsustainable in the long term.

## 3. Microcontroller Framework Design

This section focuses on developing a framework for the efficient application development of microcontrollers. This framework attempts to implement architectural principles from the higher-level programming languages. All the principles are language agnostic, but in order to implement a particular framework on a microcontroller, it is necessary to choose the language in which the framework will be implemented.

Since our framework aims to target the largest possible portfolio of potential programmers, it is advantageous for us to program a given framework using C/C++ with appropriate constraints on project configuration. This choice will make the framework usable on a large number of devices. At the same time, it is also possible to natively use already programmed libraries that support communication with sensors, actuators, and other hardware modules. Since SOLID principles are a fundamental pillar of any modern framework and are essential for creating clean, extensible, and sustainable code, we will focus on implementing these principles in the context of the C/C++ language using microcontroller-based application development.

### 3.1. SOLID Principles on Microcontrollers

As mentioned in the previous section, SOLID is an acronym for five design principles for implementing an object-oriented approach to help us write maintainable and less error-prone applications. We can satisfy almost all of the SOLID principles by choosing to implement the language using C/C++ (mainly using C++, which is an object-oriented extension of C) [16]. Nevertheless, we will focus on some of the principles in more detail and show why we chose the implementation. The first principle we will describe in this way is the interface segregation principle.

### 3.2. Interface Segregation Principle

**Figure 1:**
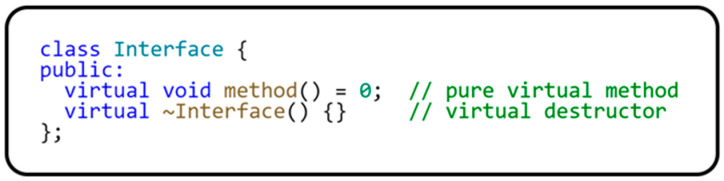
Interface implementation in C++ language.

The reason we will discuss this principle in more detail is that the C++ language does not natively support interface. When an interface is to be created in C++, it is based on an implementation of an abstract class that has one or more purely virtual methods. An example of an interface implementation in C++ is shown in Figure 1.

**Figure 2:**
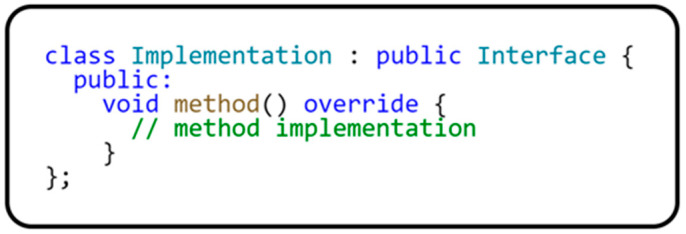
An example of using the interface.

As you can see from the code, a virtual destructor must be implemented for each interface (Interface) created in C++ to ensure proper resource release when the instance of the class implementing this interface is removed. At the same time, all methods that we want to implement in a derived class must always be defined as purely virtual. The class that defines the interface is created in the following code sample in Figure 2.

In programming practice, it is recommended that the name of the interface (Interface) start with the letter “I”. Another rule is to avoid multiple inheritance, as the “Diamond Problem” mentioned in [17] can occur. For this reason, it is better to prefer composition over inheritance.

### 3.3. Dependency Inversion Principle

Another principle we will describe in detail for implementation within the microcontroller framework is the inverse dependency principle. The dependency injection (DI) container is a particular variant of the inversion of control (IoC) principle implementation. This principle provides us with an inversion of the standard control flow in a program. This principle focuses on inverting the standard program control so that the framework or container controls the program flow instead of the traditional main program. In the context of design patterns, IoC is often implemented through specific patterns such as dependency injection or service locator. Dependency injection is a design pattern where dependencies are injected into objects instead of objects creating or finding their dependencies. Implementing a DI container that supports basic injection types such as constructor injection or method injection can be quite resource-intensive because it requires object lifecycle management in a microcontroller environment, where resources are very limited, and every byte of memory matters; such an implementation may be inappropriate or inefficient. Microcontrollers typically run simple, dedicated tasks where dynamic dependency management and the emergence of late binding can be a burden rather than a benefit.

Another design pattern that follows the IoC principle is the service locator. The service locator implements IoC by having objects obtain their dependencies from a central registry instead of injecting and managing the object lifecycle as in DI containers. Using this approach, we can optimize resource utilization, making this pattern more suitable for implementation in a microcontroller environment. To optimize the implementation of this pattern, using both memory and computational performance, we will not use run-time-type information (RTTI) in the implementation [18,19,20]. RTTI is a mechanism that allows us to obtain data-type information at run-time. While RTTI is useful in standard programming, its use is often inappropriate for microcontrollers for several reasons:

- Memory consumption: RTTI can significantly increase our program’s memory requirements because it stores additional information about data types.
- Performance: Searching and manipulating RTTI can be time-consuming and cause problems when the code requires a fast response.
- Portability and compatibility: Not all compilers and platforms support RTTI, which can cause portability and compatibility issues for our code.
- Complexity: Using RTTI can increase our code’s complexity, leading to bugs and more difficult maintenance.
- Deterministic behavior: In resource-constrained environments, such as microcontrollers, it is often crucial that the behavior of the system is predictable. RTTI can cause non-deterministic behavior, which is unacceptable.

**Figure 3:**
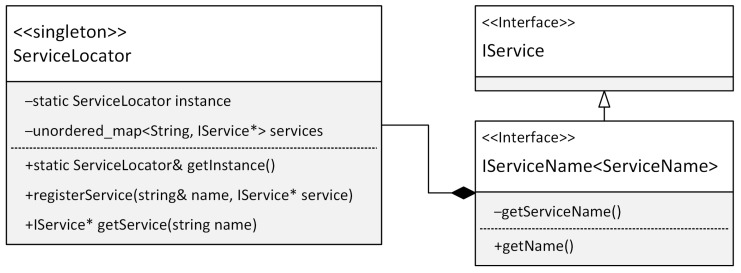
Class diagram of the ServiceLocator pattern implementation.

**Figure 4:**
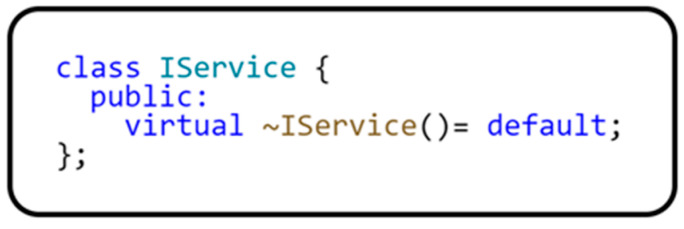
Implementation of the IService interface.

Therefore, when programming on microcontrollers, static typing and other techniques that do not require additional resources or reduce the complexity of our code are preferable. Using these assumptions, we have designed our own class implementation based on the service locator design pattern adapted to the constraints of microcontrollers. Figure 3 shows a class diagram of the service locator pattern implementation that uses the primary IService interface. This interface implements any service or object that can register to the ServiceLocator class. The implementation of this interface is shown in Figure 4.

It is also necessary to implement a virtual destructor in C++ to ensure proper resource release when a class instance is removed.

**Figure 5:**
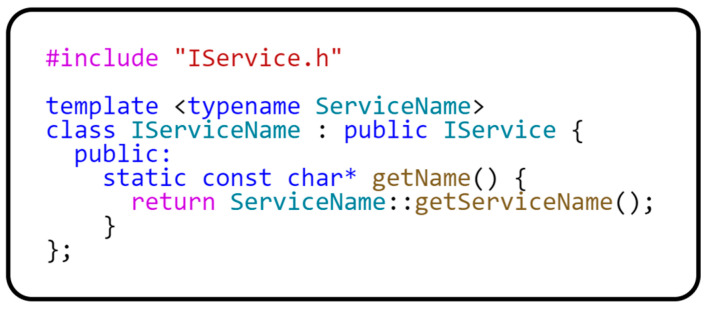
Implementation of the CRPT pattern.

Because we do not want to use the RTTI mechanism in our implementation of the ServiceLocator class and we need to uniquely identify the type of the object, another interface is created that implements the getName() method, which will store the unique identification of the object in string form. The source code for this interface is provided in Figure 5.

We must use the curiously recurring template pattern (CRTP) to implement this interface. This is an advanced design pattern in C++ that can significantly improve the code’s performance and flexibility. We will now introduce this design pattern in more detail.

### 3.4. Curiously Recurring Template Pattern

**Figure 6:**
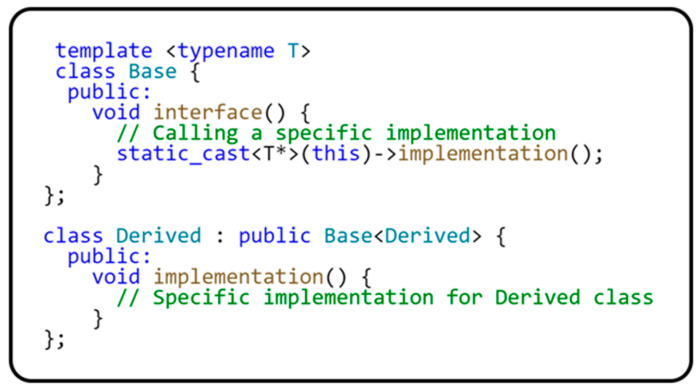
CRPT pattern usage example.

This is a design pattern that allows for static polymorphism by having the derived class inherit from a template base class to which it passes itself as a template parameter. This approach provides base class elements to behave differently for different derived classes without needing virtual functions [21]. Figure 6 shows an example of CRTP implementation in simplified form.

In this example, the Base template class expects Derived classes (such as Derived) to implement the implementation() method. When the Base::interface() method is called on an instance of the Derived class, the Derived::implementation() construct is used. Table 1 describes the advantages and disadvantages of implementing the CRTP design pattern.

**Figure 7:**
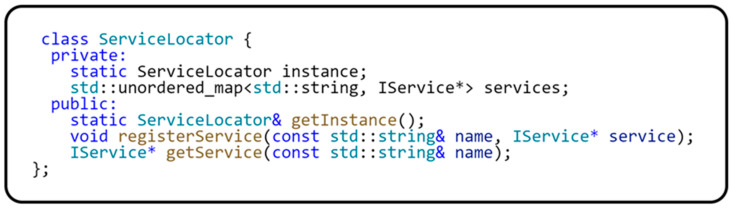
ServiceLocator implementation.

Now that we have described and defined each interface, we can describe the implementation of the ServiceLocator class. To make it convenient to work with the ServiceLocator class, we have implemented the static method getInstance(). This method always returns a single instance of the ServiceLocator class because the singleton design pattern is implemented here. To access the IoC container, the registerService(…) and getService(…) methods are implemented to set or obtain the object registered in the IoC container. The IoC container used here is a dictionary-type structure whose key is provided by the IServiceName interface implementation. The sample code for the header file implementation of the ServiceLocator class is shown in Figure 7.

**Figure 8:**
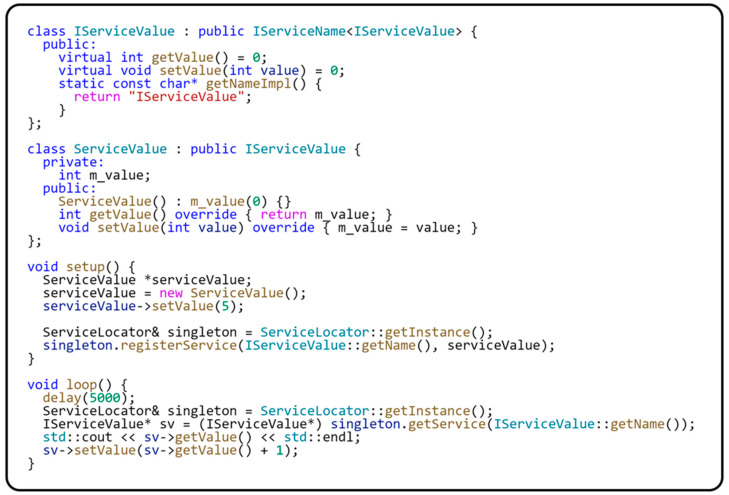
An example of working with ServiceLocator.

The following code sample in Figure 8 shows the practical use of the ServiceLocator class. In this way, we are already fulfilling the SOLID principles described in the previous section.

At the beginning of the sample example, we create an IServiceValue interface for the service we want to register in the ServiceLocator class. We then implement this interface in the IServiceValue class. In our program’s setup() method, we create an instance of the ServiceValue class and initialize the private value m_value for this instance using the setValue(…) method to the selected value. We then register this instance to the ServiceLocator class. The loop() method then shows how to access the registered instance of the ServiceValue class through the ServiceLocator class using the Iservice value interface. When the program is run, a listing will be displayed in the console that starts with the initialized value; in our example, it is the value 5. This variable is then incremented by the value 1. The value is dumped to the console on each pass of the loop() method.

We have now shown that SOLID principles can be implemented to support the development of applications on microcontrollers. In the next section, we focus on implementing a generic framework that should increase the maintainability, testability, and extensibility of these applications.

## 4. Framework for Application Development on Microcontrollers

When designing a framework for developing applications on microcontrollers, it is essential to emphasize modern software engineering principles. This framework should be based primarily on SOLID principles and design patterns. These aspects are necessary to create an environment that is robust and efficient, well-maintainable, and adaptable to future needs and changes. These principles and practices include:

- SOLID principles: The framework should be built on SOLID principles, which are the basis for creating quality, extensible, and maintainable software. This approach ensures that each part of the framework is well-defined, has clear responsibilities, and is easy to modify without intervening in existing code.
- Design patterns: Design patterns provide proven solutions to common programming problems. Using them within the framework helps create code that is not only more efficient and readable, but also easier to test and maintain.
- Modularization and extensibility: The framework should be highly modularized, making it easy to add, remove, or modify features. This modularization promotes extensibility and flexibility, allowing developers to tailor the framework to specific project needs and ensuring that individual components can be updated independently.
- Support for different microcontrollers: The framework should provide broad support for different types of microcontrollers and allow developers to work with their preferred devices and platforms. This includes abstracting hardware differences so that the same code can be used with different microcontrollers.
- Testability: A high level of testability is essential to ensure the quality and reliability of applications. The framework should support and facilitate the creation of complex test scenarios, including unit tests and integration tests. This includes simulation and mocking tools that enable effective testing in different environments.
- Maintainability and robustness: The emphasis on maintainability ensures that the framework and the applications developed using it will be easy to update and maintain in the long term. This includes a clean architecture, clearly defined interfaces, and easy maintainability and extensibility of existing features.

This approach to framework design ensures that the development of applications on microcontrollers is not only efficient and flexible but also robust, secure, and maintainable in the long term. In the previous sections, we discussed SOLID principles and design patterns, and now we will focus on implementing application modularization support.

### 4.1. Modularization and Extensibility

**Figure 9:**
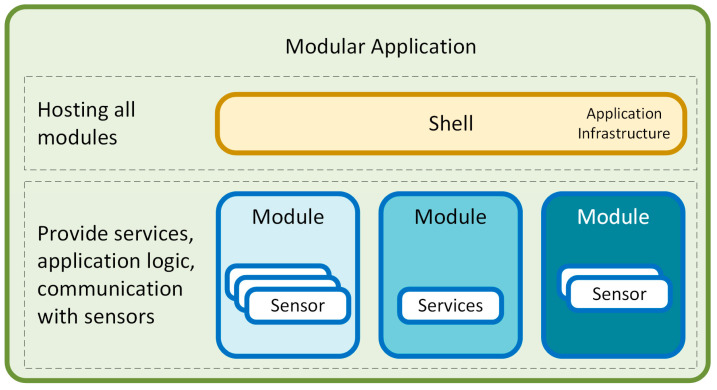
Block diagram of a modular application.

Modularization within this framework should involve the development of separate functional units and modules that are independent but can communicate freely with each other. Each module should contain some part of the application’s functionality; this can be application logic, communication with sensors, or infrastructure services. This approach facilitates the application’s development, testing, deployment, and maintenance, as each module can be developed and tested independently while contributing to the overall functionality of the system. Figure 9 shows a block diagram of a modular application.

Incorporating the idea of modular application development will significantly improve the overall structure and management of our project. There are several benefits to a modular approach to application development, as follows:

- Separation of functions: A modular design separates the application into different functional areas, making it easier to develop and test each part independently.
- Flexibility and extensibility: Modular applications are more flexible and more accessible to extend. These features make it easier to add new features and adapt to changes. This is particularly advantageous when changing from one sensor to another, with minimal impact on other system parts. Thanks to the modular architecture, a sensor can be replaced or upgraded without requiring extensive modifications to the entire application. Replacing a sensor module does not affect the integrity of the other modules, greatly simplifying the hardware upgrade process while maintaining system stability.
- Maintainability: The modular design makes the application architecture more maintainable because each module encapsulates specific functionality and is integrated through clear but loosely coupled communication channels.

### 4.2. Module as a Basic Building Block of the Application

**Figure 10:**
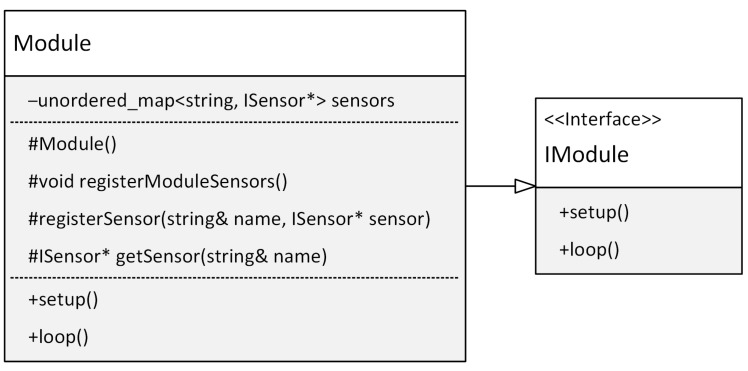
Class diagram of the Module implementation.

The basic building block of a modular application is the module. A module is a set of functions and resources organized to enable independent development, testing, deployment, and integration into a broader software system. This approach is based on object-oriented programming principles such as abstraction and encapsulation. Encapsulation is essential for maintaining the integrity and independence of modules and allows for the application’s high level of modularity and extensibility. Within each module, a central class is responsible for integrating the module into the overall system. This class implements the IModule interface, which provides a standardized method of managing modules within the application. The key methods of the IModule interface are setup() and loop(). These methods are used to manage the module’s life cycle. The IModule interface was inspired by the Arduino platform’s programming approach, which uses a similar structure with setup() for initialization and loop() for normal program execution. Therefore, the setup() method within the module is used to initialize and set it up, while the loop() method is for the ongoing execution of its functions. This modular approach allows for better code organization, simplifying code maintenance. To facilitate the implementation of modules, our framework provides a base class Module that simplifies module development. Figure 10 shows the class diagram for an application module.

**Figure 11:**
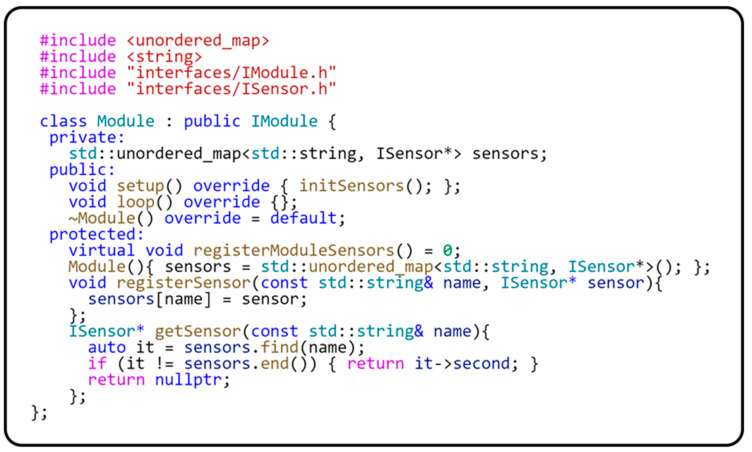
Base class Module implementation.

As can be seen in Figure 10, the Module class, in addition to implementing the setup() and loop() methods, also implements the protected methods for registering the abstract sensor registerSensor(…) and the getSensor() method for obtaining the registered sensor. Furthermore, the Module class provides an abstract method registerModuleSensors(), which must be implemented in derived classes. This method is used to register specific sensors for a given module. The section on ensuring the platform’s independence from the framework will provide a detailed description of the abstract sensor implementation. The registration of abstract sensors uses the exact mechanism applied in implementing the ServiceLocator class. The ServiceLocator class has previously been described in detail. The following code in Figure 11 shows the implementation of the Module base class, which is used to implement any module within our framework.

**Figure 12:**
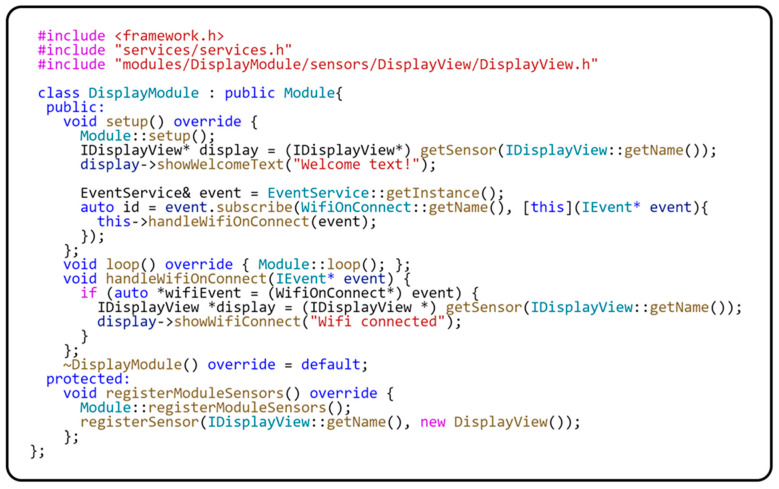
Sample implementation of a specific module.

Encapsulating all operations with abstract sensors within the module ensures that these sensors are only used in that module’s context. This approach increases modularity and ensures that each module operates with its sensors independently of other application parts. The following code sample in Figure 12 provides a clear view of how a module isolates and manages its sensors in practice, demonstrating the effectiveness of encapsulation in modular programming.

As you can see from the code sample, it is clearly specified what and in what part of the module needs to be initialized. The registration of all abstract sensors for the module uses the registerModuleSensors() method. These sensors are then initialized using the setup() method. In this method, it is also possible to log the events to which the module should respond. The loop() method then focuses on executing the module’s main function. Often, it also publishes the events that the module provides. Since the functionality of a module is encapsulated, it is necessary to implement a communication interface for two or more modules to communicate with each other. The next section will describe a more detailed description of the possible communication interfaces between modules.

### 4.3. Communication between Modules

Although the modules should be weakly interconnected, it is common for them to communicate with each other. There are several patterns for weakly coupled communication, each with its strengths. Usually, different design patterns are combined to create the final solution. The following are some of them:

- Loosely coupled events represent a sophisticated communication mechanism in software architecture where low interdependency between modules is emphasized. This concept allows a module to broadcast the information that an event has occurred while others can subscribe to these events and be informed of their occurrence. This method is based on the publish–subscribe design pattern, which is widely used for efficient and dynamic communication between different system parts. The main advantage of loosely coupled events is their ability to provide a simple yet robust form of communication. They are easy to implement and allow modules to respond to events independently of other application parts. While they offer many advantages, it is crucial to be aware that a design that depends solely or too much on events can lead to complications in maintainability and code readability. In cases where many events need to be coordinated to accomplish a single task or when a stronger form of interaction is required, it may be a better choice to consider implementing shared services.
- Shared services: These represent a fundamental concept in advanced software design, especially in the context of creating modular and scalable applications. This approach allows the decoupling of specific service implementations from their abstract definitions, providing a way to share functionality across different parts of an application. A shared service is defined as a class that offers some functionality and is accessible through a common interface. This interface abstracts away the specific service implementation, allowing applications to use these services without direct dependence on their internal details. The most common representatives of shared services include, for example, a logging or configuration service.
- Shared resources: These are another representative of communication in the architecture of modular applications, providing indirect communication between modules. This concept allows modules to share information and data through external storage, thus avoiding direct interdependencies. In a microcontroller environment, shared resources can include shared memory (EPROM), peripherals (SD card), or communication channels (Web services, MQTT). Thus, shared resources represent a flexible and efficient way of implementing indirect communication between modules in traditional software applications as well as in specific microcontroller-based applications.

In the context of communication between modules, it is important that each module maintains its independence and flexibility. Loosely coupled events allow modules to react to changes without needing tightly defined dependencies. Shared services provide a centralized functionality for different modules, simplifying application design and maintenance. Shared resources are helpful when direct communication between modules is inappropriate or difficult and allow modules to share data and state through common resources. These patterns of communication between modules help create resilient and scalable software architectures. We now provide concrete implementations of these communication patterns within our framework.

#### 4.3.1. Implementation of Communication Using Loosely Coupled Events

**Figure 13:**
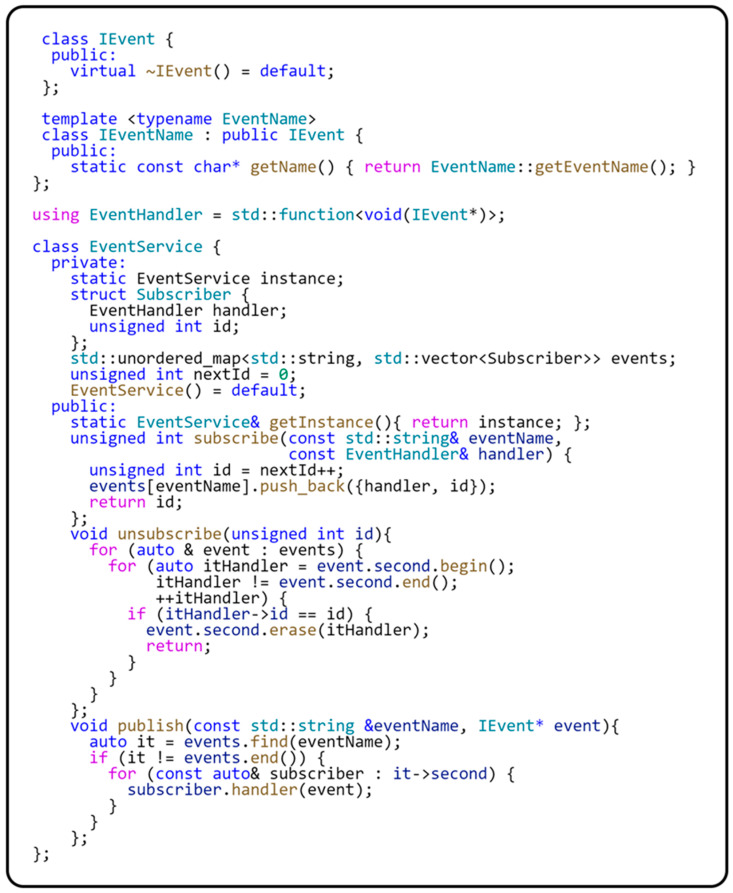
Implementation of the EventAggregator design pattern in EventService.

Loosely coupled event processing in software engineering, implemented using the publish–subscribe design pattern, allows objects (publishers) to broadcast events without the knowledge of their receivers (subscribers), who can subscribe to these events and be notified of their occurrence. This model enables flexible and efficient communication within software systems, increases application resilience to change, and supports the dynamic interaction between different modules. In our framework, we chose to implement the event aggregator design pattern to manage these events more efficiently within the structure of a modular application. This pattern provides a centralized solution for collecting and distributing events, reducing direct dependencies between components, and simplifying the overall complexity of the application. It is ideal for creating cleaner, more sustainable, and testable modular systems. The event aggregator acts as a bus that receives events from various sources and distributes them efficiently to the logged-in components, allowing for the better control and management of events in the application. By taking this approach, we have achieved significant flexibility in our development process and streamlined event management, which brings significant benefits to our framework’s overall architecture and functionality. The following code in Figure 13 shows how the Event Aggregator design pattern is implemented.

**Figure 14:**
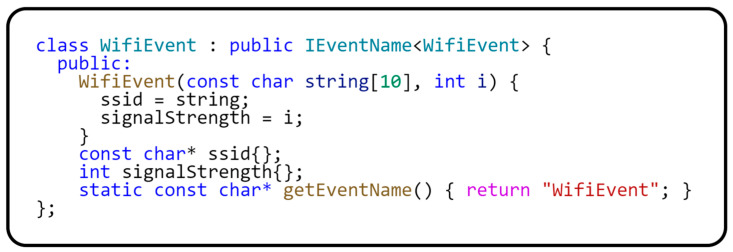
Sample event declaration for EventService.

Interface IEvent is an abstract base class for all events. It is used here as a common ancestor to define any event. Interface IEventName is a template class that extends IEvent while allowing us to identify the uniqueness of each event. This property is essential for locating the event within the event aggregator pattern. The IEventName interface implements the curiously recurring template pattern, the benefits of which were described earlier in this section. The EventHandler type is defined as std::function<void(IEvent*)>. The std::function template class wraps any callable object (such as functions, lambda expressions, function objects, and object methods) with a specific function signature that includes the given return value and parameter types. In the context of our event aggregator implementation, the std::function<void(IEvent*)> method allows us to define different means of event processing, which is essential for the flexibility and extensibility of this pattern. The subscriber can assign any function or lambda expression that matches this type to handle an event whenever it is published. The EventService class implements the event aggregator design pattern. It contains methods for subscribing to events (subscribe), unsubscribing from events (unsubscribe), and publishing events (publish). The events are managed using a map where the key is the event name, and the value is the list of subscribers. The following code in Figure 14 shows how easily you can implement a class representing an event.

**Figure 15:**
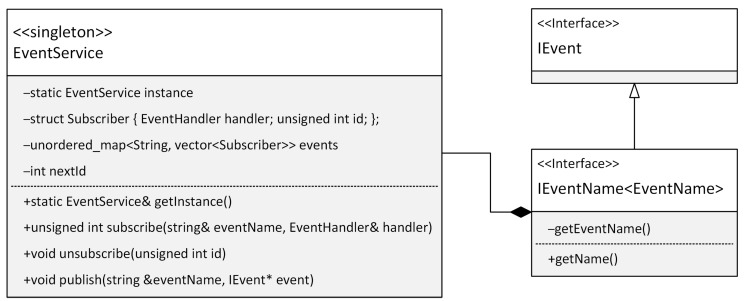
Class diagram of the implementation of the EventService class.

This class represents a specific type of event within the event system. As you can see in the code, the above class implements the IEventName interface, which defines a unique WifiEvent identifier for the event. The communications between subscribers and the event publisher are routed through this identifier. Figure 15 shows a class diagram of implementing the event aggregator design pattern.

This implementation is designed with limited microcontroller resources in mind. The memory footprint is minimized by implementing the singleton design pattern. Implementing this pattern ensures that all modules in the application share the same central point for event management. Another method of communicating between modules is to use shared services. We will describe the implementation of this principle in the next section.

#### 4.3.2. Implementation of Communication via Shared Services

**Figure 16:**
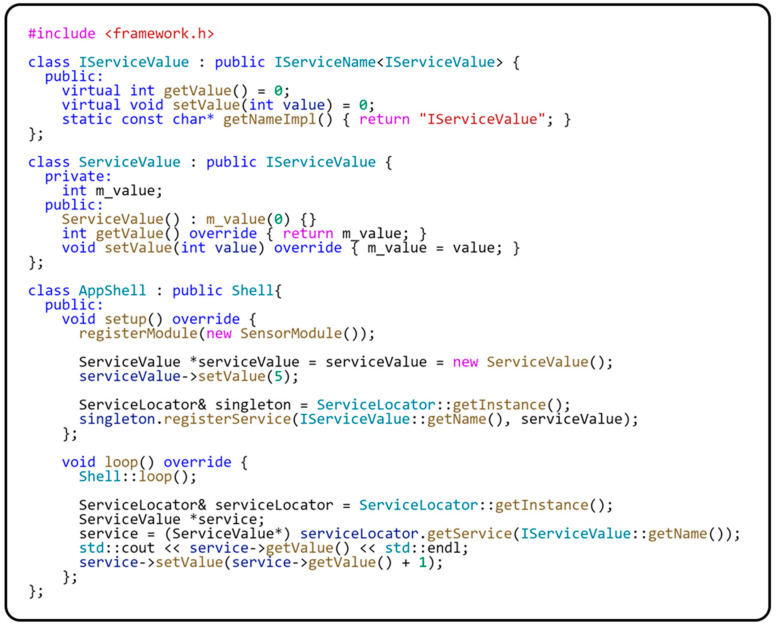
A sample implementation of a shared service.

Communication between modules through shared services is another method of interaction in modular systems. Within our framework, the ServiceLocator design pattern is used for this purpose. The section dedicated to implementing SOLID principles on microcontrollers provides a detailed description and implementation of this design. The main advantage of this approach is that it allows modules to access services and resources without the need for a hard dependency on specific implementations of these services. In this way, services can be dynamically exchanged or updated without significant impact on individual modules, greatly increasing the application’s flexibility and scalability. ServiceLocator also enables centralized service management, simplifying the system configuration and maintenance. This design pattern is particularly useful in microcontroller applications, where it is crucial to minimize dependencies and keep software complexity low while maintaining a high level of modularity and testability. The following code in Figure 16 illustrates how easily a shared service can be implemented and registered using our framework.

The code represents the implementation and use of ServiceValue through ServiceLocator in AppShell. As an abstract interface, the IServiceValue interface defines methods for handling the value and is implemented in the ServiceValue class, which stores and manages the numeric value. AppShell uses the setup() and loop() methods to register an instance of ServiceValue in the ServiceLocator. The service is initialized and registered in the setup() method, while in the loop() method, the service is retrieved from the ServiceLocator. In this method, the current value stored in the service is printed out, and this value is updated. This approach demonstrates how ServiceLocator enables the central management and easy sharing of services within a modular application, leading to flexibility in application design and reduced software maintenance costs. We have now shown an implementation of communication over a shared service, and in the next section, we will show how modules can communicate between modules over shared resources.

#### 4.3.3. Implementation of Communication over Shared Resources

In our framework, communication via shared resources is not implemented natively, which is particularly important in microcontrollers, where each resource has a significant impact. Shared resources in this environment can include peripherals such as SD cards and EPROMs or communication with external service interfaces. The access to these resources is controlled through application logic designed to respect the microcontrollers’ resource constraints and hardware capabilities. The application logic allows developers to create adapters or interfaces for specific resources, such as SD cards, that can be used for storing data or configurations. This approach enables the efficient management of these resources and provides greater control over their use while ensuring that communication with them is efficient and secure. Implementing file system functions or logging through application logic when accessing the above-shared resources makes application development easier. This approach also supports application testing and maintenance as the resources are isolated from the main application flow, which is key to developing robust and sustainable systems in a microcontroller environment.

Now that we have described how to communicate between modules with loose coupling, we move on to another critical issue in our framework: the platform independence of microcontrollers.

### 4.4. Platform Independence

Platform independence in microcontrollers is a key feature for creating applications that can run efficiently on different hardware platforms. This feature ensures that applications can be easily adapted to different types of microcontrollers without requiring significant code changes. The key to achieving platform independence is the abstraction from physical drivers and hardware specifications, which allows the application to communicate with different hardware components through a single interface. This abstraction not only simplifies development but also increases the portability of the application between different platforms.

#### 4.4.1. Ensuring Platform Independence

Now, we will see how platform independence is implemented in our framework. The key element is partitioning the application logic into individual modules, which creates the ideal environment for ensuring this independence. In implementing this principle, we have drawn inspiration from the Model-View-Controller (MVC) design pattern. While we do not directly implement this pattern, we rely on its underlying principles and philosophy. At the same time, we try to maximize the use of existing controllers for specific sensors. This approach may involve some part of the module being platform-dependent, taking advantage of existing drivers hidden behind an abstract interface. The module’s application logic then exploits this interface. This allows for a high degree of platform independence, with platform driver changes localized to a specific class or file. This approach has the advantage that any driver changes are isolated and do not affect the application’s overall functionality running on the microcontroller. It also allows the easy replacement of one type of sensor with another, including drivers, without an extensive impact on application functionality. This abstraction model facilitates the development and scaling of applications across different microcontrollers and hardware platforms. Our architecture allows developers to focus on the application logic while the details of the hardware interaction are efficiently managed in isolated components. This approach also increases code reuse and simplifies application maintenance and updates. By effectively separating application logic from hardware-dependent components, we are able to quickly respond to changes in hardware specifications and adapt our solutions to new device types. In the next section, we discuss our new Sensor-View design pattern in more detail, which is essential for implementing platform independence on the microcontroller. Sensor-View allows us to decouple the sensor logic from the user interface and other aspects of the application, which brings significant benefits in the context of platform independence. This pattern contributes to a better understanding and managing the interactions between hardware and software components and provides an effective framework for designing modular and portable microcontroller applications.

#### 4.4.2. Sensor-View Design Pattern

The Sensor-View model, which is inspired by the MVC (Model-View-Controller) architecture, can be structured to separate platform-dependent sensor controllers (Sensor) from the platform-independent interface for interacting with those sensors (View). This model allows the easy integration and replacement of sensors without modifying the rest of the application. Here is a more detailed description and design of this model:

- Sensor: Platform-dependent; each sensor driver is specific to a particular type of hardware or microcontroller. This layer handles the low-level communication with the hardware sensors, processes data from the sensors, and provides these data in a form that the application logic can further process.
- View: This layer is platform-independent and provides a consistent interface for the application to interact with sensors independent of specific hardware. The View may include logic to customize or transform sensor data to make it suitable for use in the application. Because View is decoupled from particular sensor drivers, sensors can be easily replaced or updated without changing the application logic.

**Figure 17:**
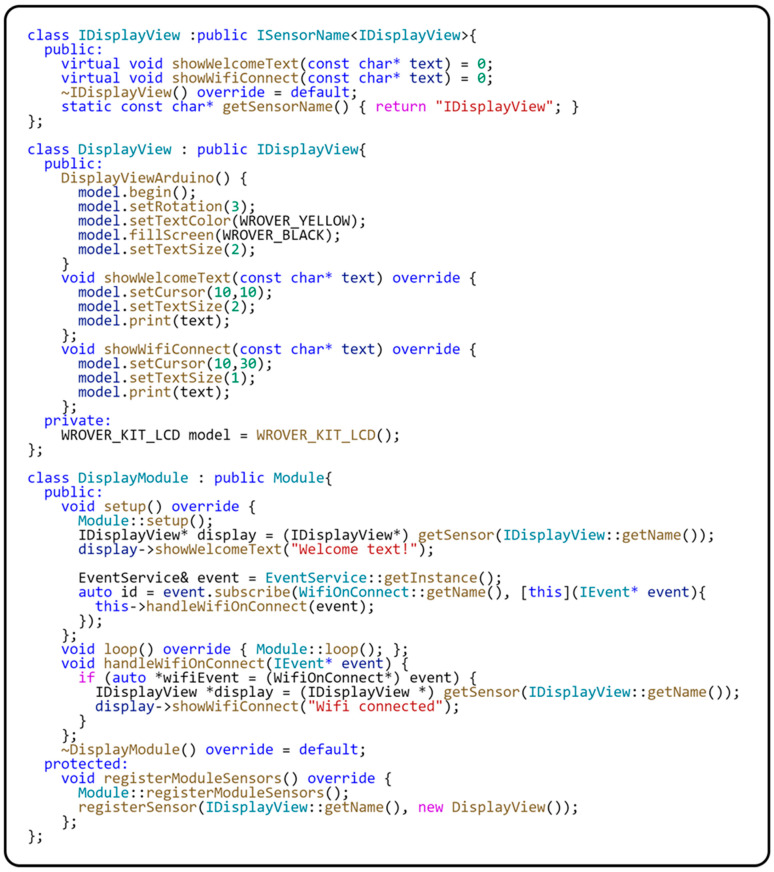
Implementation and use of the Sensor-View design pattern.

The code sample in Figure 17 illustrates the implementation of the Sensor-View design pattern and its use in a module of our framework.

The implementation of the Sensor-View design pattern is represented in our system by the IDisplayView interface and the DisplayView class. The IDisplayView interface is an abstract interface defining the showWelcomeText(…) and showWifiConnect(…) methods for displaying text on the screen. This interface serves as the basis for deriving concrete implementations of display components. The DisplayView class represents a practical implementation of this interface. This class handles all display aspects, including display initialization and display setup. In this way, the DisplayView class removes application logic from direct interaction with the hardware display, allowing for better modularity and easier code maintenance. Within the DisplayModule, the IDisplayView interface is registered for communication with the module’s application logic. This registration is completed through the registerSensor(…) method, implemented in the base class of the module. This method is responsible for registering an instance of the DisplayView class, which takes over the responsibility for physical communication with the display. This ensures the separation of hardware details from the rest of the application.

**Figure 18:**
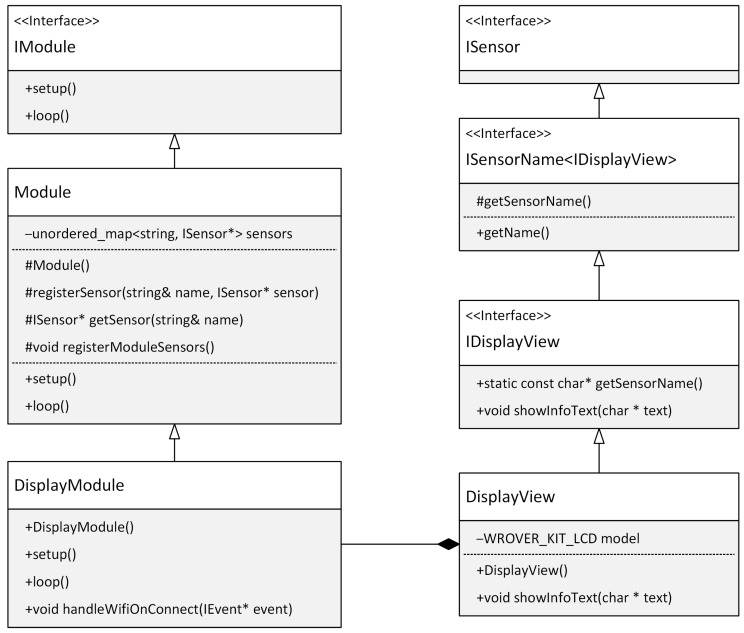
Class diagram using the Sensor-View pattern.

The Factory Method design pattern inspires the registration process, allowing the DisplayModule class to dynamically manage an instance of the DisplayView class. This arrangement provides flexibility and ease of replacing or updating display components. Thanks to this principle, the interaction between the display and the application logic is mediated through an abstract interface, resulting in increased modularity and flexibility of the overall system. The DisplayModule thus acts as a link between the specific display hardware and the platform-independent application logic. This interconnection aligns with the Sensor-View design pattern principles and strengthens our application’s platform independence. Furthermore, this model facilitates application integration and maintenance, as changes in the display driver do not affect other application parts. This allows developers to respond flexibly to new requirements or changes in the hardware environment. Figure 18 shows a class diagram of the sensor registration implementation within the module.

We have now described a module’s responsibilities, how it can interact with other modules, and how platform independence is ensured within a module. What we have not seen yet is how the application manages to work with multiple modules. We will describe this process in more detail in the next section.

### 4.5. Module Management in the Context of the Application

**Figure 19:**
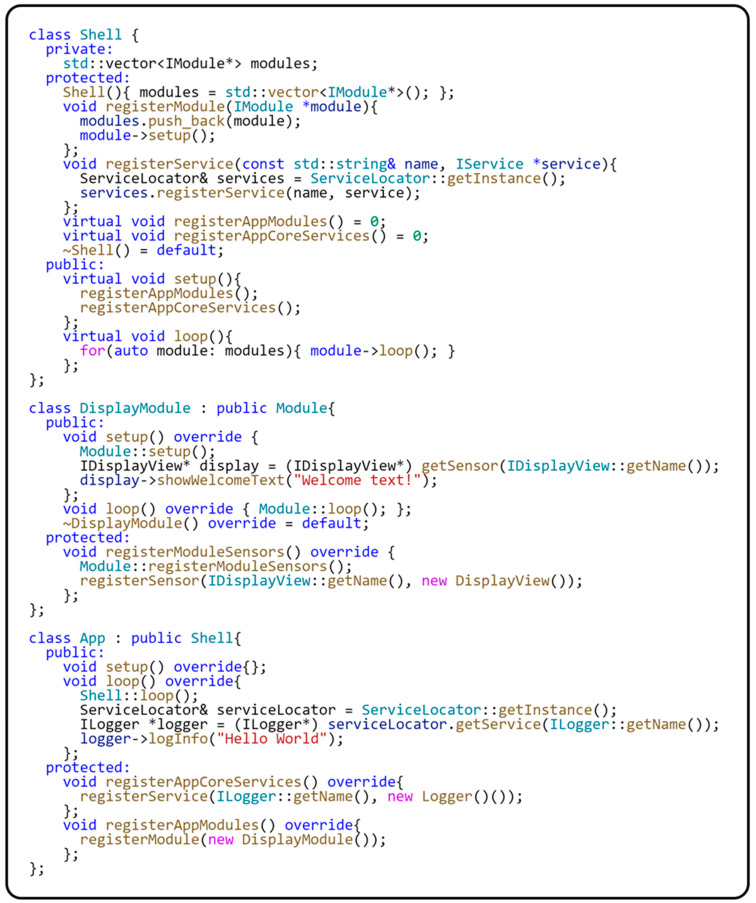
Example of using the modular framework.

When creating a modular application where the application logic is divided into several loosely coupled modules, it is crucial to ensure the effective management of these modules from the perspective of managing the entire application. This process requires a carefully designed system that allows the individual modules to operate independently while coordinating and synchronizing them so that they create a coherent overall application functionality. In previous sections, we have discussed how our framework implements critical features for supporting modular application development. We now look at how our framework handles the coordination and management of modules at the application level. The following code sample in Figure 19 demonstrates how to develop modular applications using our proposed framework.

Within our framework for modular applications, the Shell class serves as the base class for coordinating and managing the modules of an application. This class contains a list of modules used in the application, which are registered using the registerAppModules() method. This is an abstract method that must be implemented in a derived class. The modules used in the modular application are then registered using this method. The registerModule(…) method, which is implemented in the Shell class, is used to register modules. The module registration process in the Shell class uses the factory method design pattern, which allows for the flexible and dynamic addition of modules to the application. The priority and processing order of modules is determined by their registration order, which ensures the efficient management of application operation. Each registered module is automatically initialized using the setup() method. This ensures that it is ready for integration into the application. In addition to modules, the Shell class also allows the registration of infrastructure services implementing the IService interface. The registerService(…) method is used to register these services, which makes it easy to manage them using the ServiceLocator class. For clear separation of responsibilities within the Shell class, individual services are registered using the registerAppCoreServices() method. It is an abstract method that requires implementation in a derived class and is intended to register the infrastructure services of a modular application. The loop() method in the Shell class is essential for executing the logic of all modules and allows you to keep the application active and responsive. This method iterates through all registered modules, calling each module’s loop() methods. This behavior is crucial in keeping the application running continuously.

**Figure 20:**
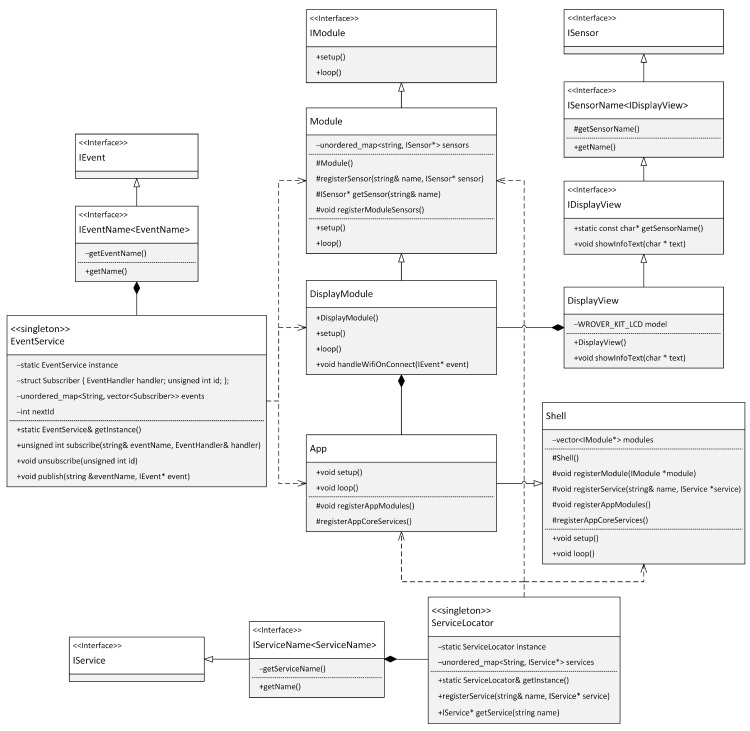
Class diagram of an application using the framework.

The app class is the input class for the modular application and is responsible for initializing the entire application. The App class is a child of the Shell base class. The registerAppCoreServices() method in the app class is used to register the infrastructure services of the modular application, and the registerAppModules() method is used to register the modules. Subsequently, the setup() method of the App class initializes the application’s default state. Within our sample code, the registerAppModules() method registers a specific application module, such as the DisplayModule class, and the registerAppCoreServices() method registers the Logger infrastructure service using the registerService(…) method. The Logger service is responsible for the general logging mechanism in our sample application. This structure of the App class allows us to efficiently manage and coordinate both modules and services within the entire application. Figure 20 illustrates the relationships between the classes and how each component is integrated within our framework.

This diagram provides a visual overview of the application structure and shows how modules, services, and other key components are connected and work together to achieve application functionality. The diagram also illustrates the inheritance hierarchy and dependencies between classes, allowing a better understanding of the architectural principles used within the modular application. A highly efficient and sustainable architecture can be achieved using the framework and applying SOLID principles during the application development process. This accessible and structured arrangement recommends having a tightly defined application structure and promotes the separation of responsibilities within the code, contributing to better modularity and sustainability.

### 4.6. Application Structure

For developing modular applications, following a specific project structure specifying where each part of the application is developed is a good idea. This structure provides:

- A clear framework for organizing the code and its components.
- Making the project easier to navigate.
- Increasing code readability.
- Aiding efficient development and maintenance.

A key element of such a structure is dividing the project into logical blocks or modules responsible for specific functionality. Each module can be developed and tested independently, allowing better source code management and the easier integration of new features. The project structure should also include centralized dependency and configuration management, which simplifies the management of libraries and tools used in the application. This approach helps to prevent dependency conflicts and facilitates project updates. Implementing and following a clear project structure ultimately leads to greater development efficiency, makes it easier to scale and extend the application, and increases its sustainability.

**Figure 21:**
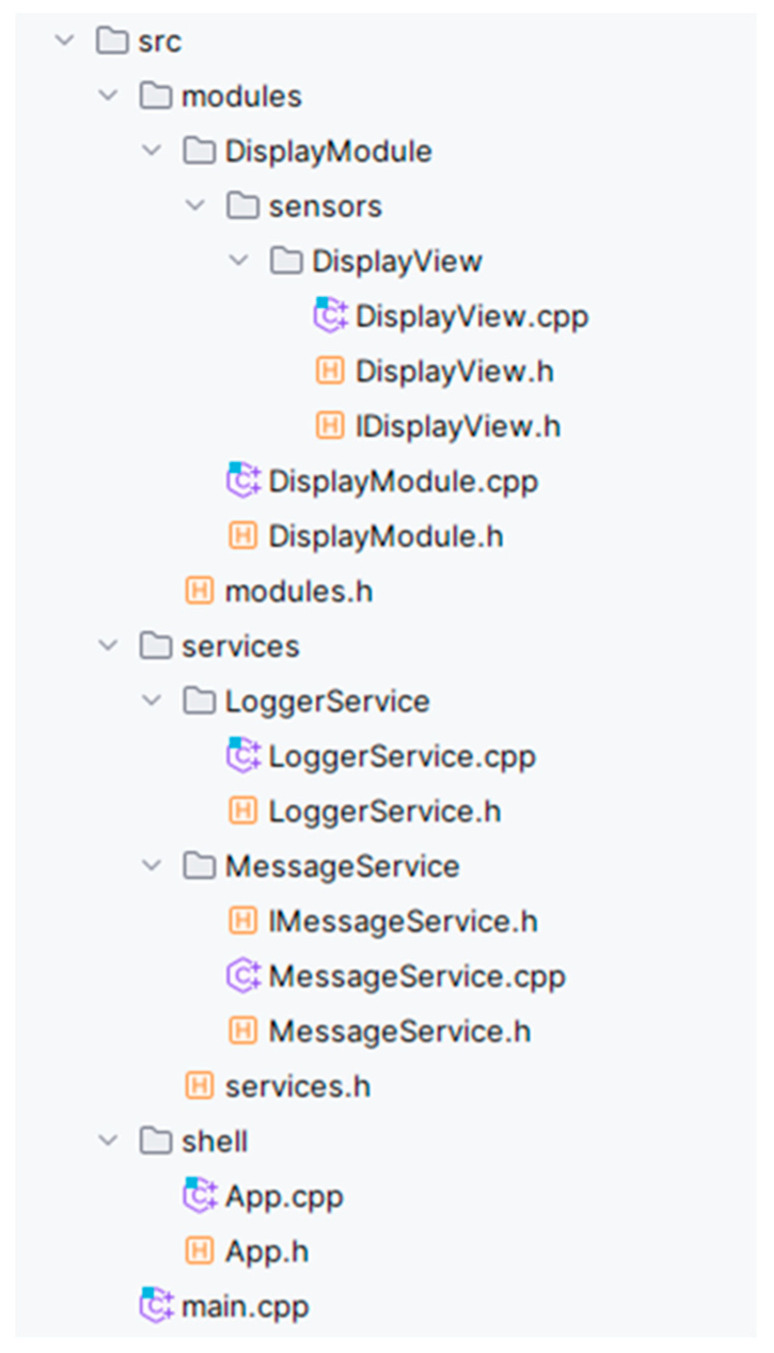
Recommended modular project structure.

Since our proposed framework will be distributed as a multiplatform library, we will not describe it in detail in the description of the application structure. The structure of our project, as shown in Figure 21, is carefully divided into three primary directories: modules, services, and shell. Within the main src directory, we also find the main.cpp or main.ino file, depending on the development environment used. The main.ino file is typical for the Arduino IDE, while the main.cpp is standard for the highly preferred development ecosystem in the PlatformIO microcontroller area. PlatformIO is known for its broad support of various development boards and microcontrollers and allows for considerable flexibility in development. The main.cpp file serves as the central entry point for the entire modular application, where the App class is initialized. This core App class is implemented in the shell directory. The App class plays the role of coordinator for the entire application, providing the registration and management of modules and infrastructure services. The App class embeds references to the services.h and modules.h header files, located in the service and module directories. These files contain references for all key services and modules in our modular application, ensuring their efficient management and easy integration into the main development flow. Another important services directory contains all the infrastructure services required for the modular application to function. Within this directory, each service is placed in its own subdirectory whose name corresponds to the name of the service. In these subdirectories, we find files that contain both the implementation and the infrastructure service interface. Note the LoggerService. Unlike the others, this service does not have its own interface file since its interface is already part of our framework. An essential element of the services directory is also the services.h header file, which includes references to all services implemented in this directory. This centralization of references greatly facilitates the management of individual services and their effective integration into the overall structure of our modular application, increases clarity, and allows easier management of dependencies between components. The last essential directory in our modular application is the modules directory, which is used to develop all application modules. Like the services directory and the modules, the .h header file is located here. This file is crucial as it contains references to all the modules developed within this directory, making it easier to manage and integrate them into the modular application. A closer examination of the modular structure reveals a specific sensor directory, which aims to separate the platform-dependent parts of the application from the application logic. Under this directory, individual sensors are located, each in its own named subdirectory. These sensors are implemented following the Sensor-View pattern described earlier in this section. Each sensor includes not only the implementation itself but also a communication interface that allows interaction with the application logic of that module.

The proposed structure of the software project, shown in Figure 21, represents a well-thought-out and systematic approach to developing a modular application. Each of the key directory modules, services, and shell has a specific purpose, and together, they form a solid foundation for flexible and scalable application development. The header files in the services and modules directories provide a centralized approach to managing and integrating components, significantly increasing code efficiency and maintainability. The sensor subdirectory in the modules directory demonstrates our ability to decouple platform-dependent components from application logic, allowing us to achieve a higher level of code abstraction and reuse. This carefully designed modular structure of our project promotes efficient development and facilitates future extensions and maintenance of the application, but also contributes significantly to the robustness of the testing processes. By dividing the application into separate modules, we can implement targeted tests for each more minor part of the system. This approach allows the detailed verification of the functionality of individual components independently of other parts of the application. This leads to an increase in the quality and reliability of the entire software solution.

Thanks to the separation of responsibilities within the application, which is implemented using our modular framework, the given application logic is easily testable using the relevant test frameworks [22]. We recommend using GoogleTest and Unity frameworks for testing [23,24,25]. A significant advantage of our framework is the ability to test application logic without direct connection to physical sensors, which is made possible by the Sensor-View design pattern and increases the efficiency of the development process.

## 5. Discussion

Our research has focused on developing a modular framework for microcontrollers that addresses the fundamental need for application scalability and sustainability. In the discussion, we will answer the essential questions that have guided our research work.

- What made us develop it?

A common problem in developing applications for microcontrollers is their limited extensibility using traditional methods, which often results in the need for a complete rewrite of the application. Increasingly, developers are turning to principles and practices from higher-level programming languages, which began to be applied more significantly in the early 2000s, to address similar problems of extensibility and maintainability. While transferring this knowledge to the microcontroller platform brings challenges associated with limited resources, it also offers new opportunities for more efficient development. In one of our previous contributions, we dealt with the design of an advanced application architecture for microcontrollers that used standard OOP principles [26]. However, the components used in this architecture had a tight connection between them, which reduced the simple maintenance and extensibility of the application. We have now set the ambition to improve the architecture by applying advanced programming techniques commonly used in higher programming languages. Our goal was to implement SOLID principles and the application of design patterns in the context of microcontrollers, which present significant challenges due to the limited resources of these devices. Although there are previous studies [27] focused on the implementation of SOLID principles in the environment of microcontrollers, they often result in only the partial application of these principles, which is mainly caused by complications with the implementation of the inversion of control (IoC) principle. IoC is fundamental for reducing complex dependencies between software components, but its application in resource-constrained environments brings specific challenges, including the need for efficient memory management. In our work, we addressed these challenges and developed a modular framework that implements design patterns while respecting SOLID principles. This modular framework is designed for use in diverse projects related to the Internet of Things (IoTs) and represents a universal, scalable solution responding to the growing demand for interconnected devices [28]. We are currently preparing an article that will introduce our framework through concrete examples that will demonstrate its application and adaptability to the specific requirements of various IoT projects. With this approach, we will demonstrate the practical applicability of our framework and provide guidance for developers seeking to implement sophisticated programming techniques in the design and development of microcontroller applications. These IoT projects, including the modular framework, are already ready on the GitHub repository and can be provided on request. Once the upcoming article describing the implementation of these projects is published, these repositories will be publicly available.

- Why use these principles?

Applying these principles leads to a structured architecture, which provides a higher level of abstraction and facilitates code reuse. This improves the management of complex dependencies between application components. Structured design further simplifies the development process for programmers, as a clearly defined architecture makes it easier to understand, develop, and extend application functionality. Individual components are designed to be as independent as possible, allowing programmers to isolate and test functional blocks without unnecessary dependencies on other system parts. This approach contributes significantly to software modularity and sustainability, which is especially crucial in the microcontroller environment where limited resources must be managed efficiently. This approach makes it possible to introduce testing independent of the hardware device, reducing the costs associated with the most expensive phase of development, which is testing on specific hardware.

- How is this advantageous?

Our framework’s modularity and testability allow independent testing of individual application modules without deploying to physical hardware components. This technique significantly reduces the application development cost because it will enable test automation and reduce the time required for testing on specific hardware. It also lowers program breakups because the error that causes them is detected very fast. In addition, the modularity makes it easy to adapt to changes in hardware and software specifications. Another can easily replace each module with equivalent functionality but which can communicate with different hardware. This approach increases the flexibility and extensibility of the system and simplifies maintenance and upgrades, contributing to the long-term sustainability and robustness of the overall software.

- Why is this approach unique and not been implemented before?

The reason no one has taken this approach before may be related to the conservative approach in microcontroller programming and the lack of experts capable of programming across different platforms and application types. There are relatively few experts who can write efficient high-level applications as well as low-level applications for microcontrollers. The traditional paradigm in embedded systems often limits innovation by emphasizing resource optimization and stability, which can lead to hesitation in choosing more sophisticated programming techniques. This conservatism can be overcome by adapting SOLID principles and design patterns in the context of microcontrollers, which have long been considered difficult to apply due to the limited resources of these devices. The approach we have chosen involves applying advanced programming techniques typically used in higher-level programming languages, which allows us to overcome the barriers between different levels of abstraction and effectively integrate these techniques into the microcontroller environment. These advances offer the opportunity to develop modular and flexibly scalable applications that are robust, easy to maintain, and extend, fundamentally changing the approach to software development for microcontrollers.

## 6. Conclusions

In our article, we have demonstrated that SOLID principles are fully applicable to microcontrollers, thereby improving both the maintainability and testability of applications. We have designed a prototype modular framework for developing microcontrollers based on these principles and using design patterns. This framework uses SOLID principles, including the dependency injection principle. In addition, we have shown how to implement this principle on microcontrollers without using the run-time type information (RTTI) mechanism, which can cause performance and memory requirement problems on some microcontrollers. To increase the efficiency of the development process, we have designed our entire framework modularly, where each module has to conform to a clearly defined structure, making the project easier to maintain. At the same time, within the framework, we addressed how to decouple the application logic from the physical implementation over the drivers of each sensor. This separation of responsibilities allowed us to define a new design pattern for Sensor-View. This design pattern guarantees platform independence in our framework and will enable us to work with sensors within individual modules. We also designed a mechanism for communication between loosely coupled modules. The modular application development leads to greater flexibility and easier application extensibility. Based on the clearly defined rules of module development, we designed an application class structure that serves as the program’s entry point and manages the modules within the application. Thanks to these decisions, our framework has a clearly defined design structure that simplifies the development of modular applications.

We have also implemented proven principles into our modular framework based on the experience gained from the Arduino and ESP-IDF frameworks. In the next development phase, we will focus on implementing the real-time operating system (RTOS) interface. This interface is crucial for highly reactive and reliable asynchronous data processing applications. Implementing an RTOS will allow our framework to manage tasks and processes more efficiently, thereby increasing overall reactivity and reducing latency when processing real-time input and output. In parallel, we plan to extend our modular framework to support the Rust programming language. This initiative aligns with the latest development trends and offers significant security and efficiency benefits. The Rust implementation should increase the safety of our solution thanks to its memory management system, which effectively prevents common errors such as memory overflows and race conditions.

## Figures and Tables

**Table 1:** Advantages and disadvantages of the CRTP design pattern.

Advantages	
Performance optimization	Eliminates the overhead of virtual calls.
Code reuse	Allows sharing of code between different derived classes.
Type safety	Types are verified at compile time; this reduces the chances of errors at run-time.
**Disadvantages**	
Complexity	It may reduce code readability, especially for developers unfamiliar with C++ templates.
Limited flexibility	Reduced code flexibility due to dynamic polymorphism not being supported. All types must be known at compile time.
Code redundancy	Using CRTP with a large number of derived classes can lead to redundancy in the compiled code because a separate method enumeration is created for each derived template type, increasing the binary’s overall size.

## Data Availability

The data and sources codes presented in this article are available on request from the corresponding author.
